# Physicians' eye-tracking in neonatal intensive care: evaluating a mechanical ventilator panel

**DOI:** 10.1590/1984-0462/2026/44/2025313

**Published:** 2026-08-17

**Authors:** Pâmella Cristina do Prado Franco, Marina Carvalho de Moraes Barros, Roberto Gonçalves de Magalhães, Simone Yumi Tsuji Assunção, Beatriz Mesquita Mello, Ana Silvia Scavacini Marinonio, Mandira Daripa Kawakami, Milton Harumi Miyoshi, Maria Fernanda de Almeida, Tatiany Marcondes Heiderich, Rafael Nobre Orsi, Carlos Eduardo Thomaz, Ruth Guinsburg

**Affiliations:** a Universidade Federal de São Paulo, Escola Paulista de Medicina, Department of Pediatrics, Division of Neonatal Medicine, São Paulo, SP, Brazil.; b Centro Universitario FEI, Department of Electrical Engineering, Image Processing Laboratory, São Bernardo do Campo, SP, Brazil.

**Keywords:** Infant, newborn, Intensive care units, neonatal, Ventilators, mechanical, Eye-tracking technology, Clinical decision making, Recém-nascido, Unidades de terapia intensiva neonatal, Ventiladores mecânicos, Tecnologia de rastreamento ocular, Tomada de decisão clínica

## Abstract

**Objective:**

Assess physicians’ visual attention when evaluating a mechanical ventilator panel to decide whether to adjust ventilatory parameters in a newborn on mechanical ventilation.

**Methods:**

Experimental study involving physicians caring for critically ill neonates. Three clinical scenarios related to arterial blood gas abnormalities (hypoxemia, hypercarbia or normal blood gases) were presented on a computer screen, followed by a 30-second video of a ventilator panel while eye-tracker recorded their gaze. Three areas of interest (AOI) were defined: monitored parameters, adjusted parameters, curves and loops. Visual attention was analyzed in the first 10 seconds of the video. Outcomes included visual fixation (Yes/No), number, and duration (seconds) of visual fixations on each AOI. Multivariate analysis of variance was used to compare outcomes among AOI.

**Results:**

Seventy-five physicians participated, with ocular signal capture acquisition in 95±5% of the time. All physicians (100%) fixated their gaze on the curves/loops, 95% on monitored parameters and 80% on adjusted parameters. Visual fixations were more frequent and longer for curves/loops (8.03±5.98; 2.07±1.61sec), followed by monitored (5.40±5.00; 1.21±1.29sec) and by adjusted parameters (14.72±7.64; 3.71±2.12sec). No differences were observed across clinical scenarios. Physicians without specialization in neonatology or pediatric intensive care focused more on adjusted parameters than specialists.

**Conclusions:**

When evaluating a ventilator panel to decide respiratory management, physicians focused more on the curves and loops, regardless of the clinical scenario. Physicians without specialized training in neonatology or pediatric intensive care had greater visual fixation on adjusted parameters of the ventilator panel than specialists.

## RESUMO

**Objetivo:**

Verificar a atenção visual de médicos ao avaliarem um painel de ventilador mecânico para decidir se os parâmetros ventilatórios devem ser ajustados em um recém-nascido.

**Métodos:**

Estudo experimental envolvendo médicos que cuidam de neonatos enfermos. Três cenários clínicos relacionados a anormalidades dos gases sanguíneos arteriais (hipoxemia, hipercapnia ou gases sanguíneos normais) foram apresentados em tela de computador, seguidos por um vídeo de 30 segundos de um painel de ventilador, enquanto o olhar era rastreado. Três áreas de interesse (AOI) foram definidas: parâmetros monitorados, parâmetros ajustados, curvas/*loops*. A atenção visual foi analisada nos primeiros 10 segundos do vídeo. Os desfechos incluíram fixação visual (Sim/Não), número e duração (segundos) das fixações visuais em cada AOI. Análise de variância multivariada foi utilizada para comparar os desfechos entre as AOI.

**Resultados:**

Setenta e cinco médicos participaram, com aquisição do sinal ocular em 95±5% do tempo. Todos os médicos (100%) fixaram o olhar nas curvas/*loops*, 95% nos parâmetros monitorados e 80% nos ajustados. As fixações visuais foram mais frequentes e mais longas nas curvas/*loops* (8.03±5.98; 2.07±1.61s), seguidas pelos parâmetros monitorados (5,40±5.00; 1.21±1.29s) e ajustados (14.72±7.64; 3.71±2.12s). Não houve diferença entre os cenários clínicos. Médicos sem especialização em neonatologia ou terapia intensiva pediátrica concentraram-se mais nos parâmetros ajustados do que os especialistas.

**Conclusões:**

Ao avaliarem um painel de um ventilador para decidir sobre o manejo respiratório, os médicos concentraram-se mais nas curvas/*loops*, independentemente do cenário clínico. Médicos sem treinamento especializado apresentaram maior fixação visual nos parâmetros ajustados do painel ventilatório do que os especialistas.

## INTRODUCTION

Respiratory failure, particularly in preterm infants, is the main cause of admission to neonatal intensive care units (NICU).^1,2^ Preterm births are frequent all over the world. In the USA, in 2022, 10.4% of live births were preterm, 0.6% had less than 28 weeks’ gestation, 0.9% between 28–31 weeks, and 8.8% between 32–36 weeks.^3^ In a cohort of 373,167 live births from Australia and New Zealand in 2018, among infants less than 32 weeks’ gestation, 49% required mechanical ventilation for a median of eight days, as well as 24% of those between 32–36 weeks for four days.^4^ Inadequate respiratory management in the neonatal period is associated with complications such as bronchopulmonary dysplasia and brain injury,^5,6^ in addition to prolonged hospital stay.^7^

The decision to adjust ventilatory parameters is a complex process influenced not only by the physician's knowledge of the disease and the newborn's clinical condition, but also by the interaction with the ventilator interface. In a study involving 137 adult patients on mechanical ventilation, 69% of them experienced at least one critical incident related to tracheal intubation, monitoring of mechanical ventilation, or during ventilatory weaning and extubation.^8^ Despite this finding, we did not find studies evaluating the interaction of health care professionals with ventilator interfaces in the neonatal setting.

Identifying healthcare professionals’ visual attention when evaluating a ventilator panel and deciding on adjustments to a newborn's respiratory support can provide insights into their interaction with the ventilator interface and enhance training programs.^9^ Eye-tracking technology enables the identification of specific areas of the ventilator panel that attract their attention during parameter adjustments.^10^

The aim of this study was to assess physicians’ visual attention when evaluating a mechanical ventilator panel to decide whether to adjust ventilatory parameters in a newborn on mechanical ventilation. As secondary aims, this study examined the differences in physicians’ visual attention when presented with clinical scenarios of hypoxemia, hypercapnia, and normal blood gases, and compared the visual attention patterns of physicians with or without specialization in neonatal/pediatric intensive care. Our hypothesis was that physicians assessing ventilator panels in neonatal intensive care do not consider all available data when presented with multiple pieces of information.

## METHOD

An experimental study was conducted at the Neonatal Unit of Hospital São Paulo, the University Hospital of Escola Paulista de Medicina, Universidade Federal de São Paulo, from February to August 2022. The project was approved by the institution's Research Ethics Committee under Certificate of Presentation for Ethical Appreciation (CAAE) No. 52924421.0.0000.5505. After explaining the steps of the experiment, which consisted of obtaining sociodemographic data and observing an image and video of a ventilator panel on a computer screen, during which the gaze would be tracked, written informed consent was obtained from all participants.

Physicians who had previously cared for newborns in intensive care, such as pediatricians, neonatologists, and pediatric intensivists, as well as pediatric residents, were invited to participate in the study. Physicians with profound visual impairment and those for whom it was not possible to calibrate the visual tracking equipment were excluded. After the experiment, those with ocular signal acquisition for less than 70% of the experiment time were excluded.^11-13^

The study was carried out in a closed room, with artificially controlled light, where the participant was ergonomically accommodated in a chair and positioned in front of the monitor of the visual tracking equipment. The equipment used was the Tobii model TX300 (Tobii Technology AB, Danderyd, Sweden), which consists of a 23-inch monitor with an infrared lighting system, imperceptible to the human eye, which creates a marker by reflecting on the cornea. Two cameras located at the bottom of the equipment, with a data acquisition frequency of 300Hz, detect eye movement and record the individual's gaze fixations.

Demographic data and data regarding graduation, specialization, and professional experience in Neonatal ICU or Pediatric ICU were obtained through interviews, as well as experience with the iX5^®^ ventilator (Vyaire Medical, Chicago, Illinois, USA). The participants’ anxiety was assessed using the Beck Anxiety Inventory, which evaluates the presence and the intensity of 21 anxiety symptoms in the previous 15 days (0=absent, 1=mild, 2=moderate, 3=intense); the overall score was classified as low (0–21 points), moderate (22–35 points), or severe (greater than 35 points).^14^

Three clinical neonatal cases were created as visual stimuli for the participants: one with hypoxemia, another with hypercapnia, and the last one with normal blood gases. The clinical case presented was: "*You are the on-call physician in the NICU, and you are evaluating a 28-week gestation newborn who is 10 hours old and weighs 1000 g. The newborn is on mechanical ventilation by tracheal tube. You are checking the ventilatory parameters on the mechanical ventilator*". The three clinical cases differed only in blood gas and ventilator parameters. Each participant evaluated only one clinical case (Table 1). Participants did not evaluate the other clinical cases, as prior exposure to the initial challenge could have influenced their responses, particularly by altering their visual attention.

**Table 1:** Adjusted and monitored ventilator parameters and blood gas analysis results for the three clinical cases in the experiment.

Adjusted parameters		Monitored parameters	Arterial blood gas analysis
Case 1: Hypoxemia			
	Flow 6 L/min	PEEP 5 cmH_2_O	pH 7.29
	PIP 18 cmH_2_O	PIP 18 cmH_2_O	pCO_2_ 50 mmHg
	PEEP 7 cmH_2_O	Inspiratory volume 3.7 mL	pO_2_ 40 mmHg
	RR 30 mpm	Expiratory volume 3.0 mL	Base excess −4,0
	Inspiratory time 0.30 seconds	RR 30 mpm	Bicarbonate 15 mEq/L
	FiO_2_ 0.30	FiO_2_ 0.30	SO_2_ 85%
		Minute volume 0.15 L	
		Ratio volume/weight 5.1 mL/kg	
		Air leak 0%	
Case 2: Hypercapnia			
	Flow 6 L/min	PEEP 7 cmH_2_O	pH 7.17
	PIP 16 cmH2O	PIP 20 cmH_2_O	pCO_2_ 80 mmHg
	PEEP 5 cmH2O	Inspiratory volume 5.1 mL	pO_2_ 50 mmHg
	RR 40 mpm	Expiratory volume 5.1 mL	Base excess −6,0
	Inspiratory time 0.35 seconds	RR 40 mpm	Bicarbonate 16 mEq/L
	FiO_2_ 0.30	FiO_2_ 0.30	SO_2_ 91%
		Minute volume 0,13 L	
		Ratio volume/weight 3,0 mL/kg	
		Air leak19%	
Case 3: Normal blood gas			
	Flow 6 L/min	PEEP 5 cmH_2_O	pH 7.32
	PIP 15 cmH_2_O	PIP 18 cmH_2_O	pCO_2_ 48 mmHg
	PEEP 5 cmH_2_O	Inspiratory volume 5.4 mL	pO_2_ 67 mmHg
	RR 35 mpm	Expiratory volume 4.9 mL	Base excess −3,5
	Inspiratory time 0.35 seconds	RR 35 mpm	Bicarbonate 18 mEq/L
	FiO_2_ 0.35	FiO_2_ 035	SO_2_ 94%
		Minute volume 0,18 L	
		Ratiovolume/weight 4,9 mL/kg	
		Air leak 9%	

mpm: movements per minute; L/min: liters/minute; pCO_2_: partial pressure of carbon dioxide; PEEP: positive end-expiratory pressure; PIP: inspiratory pressure; pO_2_: partial pressure of oxygen; RR: respiratory rate; FiO_2_: fraction of inspired oxygen; SO_2_: oxyhemoglobin saturation.

Source: Prepared by the authors.

After calibrating the equipment, the clinical case to be presented was randomly selected using an online tool.^15^ Before the experiment began, a screen with detailed instructions for the experiment, including what would be shown on each screen, as well as the time available for reading or whether this time was free, was shown to the participants. The experiment consisted of three screens. The first screen displayed an image of the ventilator panel for 30 seconds to familiarize participants with the layout. The second screen presented the selected clinical case, including arterial blood gas results and ventilatory parameters adjusted in a time-cycled, pressure-limited (TCPL) mode, according to the case (Table 1), with no time restrictions for reading. The third screen showed a 30-second video of the ventilator panel, displaying adjusted and monitored parameters, pressure, flow, and volume curves, as well as pressure-volume and volume-flow loops, identical to those on the first screen. During this period, participants’ gaze was tracked.

**Figure 1:**
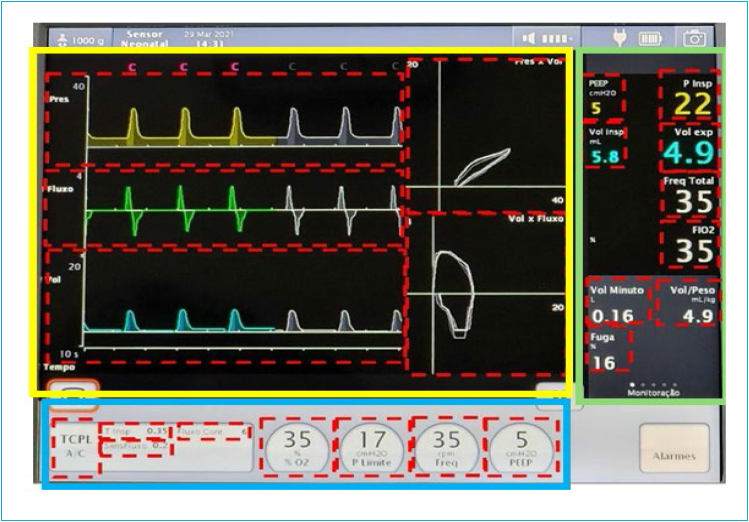
Areas of interest delimited on the mechanical ventilator panel: curves and loops (yellow); monitored parameters (green); adjusted parameters (blue).

Source: Image obtained by the researchers.

To assess the visual tracking outcomes, three areas of interest were defined on the ventilator panel image: monitored parameters, adjusted parameters, and curves and loops (Figure 1). The visual tracking outcomes were assessed during the first 10 seconds of the video in each AOI: gaze fixation (yes vs. no), number of visual fixations, and duration of visual fixations (seconds). Visual fixation was characterized by the time the eyes remained relatively still in a given place, with a movement speed of less than 30 degrees/second.^16^ The decision to analyze the ventilator video for 10 seconds was based on the rapid nature of clinical decision-making. Orsi et al.^17^ demonstrated that maximum pupillary dilation — an indicator of peak cognitive effort and, consequently, the moment of decision-making^18,19^ — occurred approximately two seconds after adults began evaluating photographs of newborns taken at rest and during a painful procedure, while judging the presence or absence of pain. As no prior studies have investigated the specific experimental paradigm proposed in our study, we extended the observation period to the first 10 seconds.

The comparison of the visual tracking outcomes of the participants during ventilator panel evaluation across the three clinical scenarios—hypoxemia, hypercapnia, and normal blood gases — was conducted using multivariate analysis of variance (MANOVA–Pilai trace), which evaluates the effect of a factor on multiple response variables simultaneously, considering the correlation between them, and adjusted for anxiety score and prior experience with the iX5^®^ ventilator (Vyaire Medical, Chicago, Illinois, USA). Similarly, the comparison of visual tracking outcomes between physicians with and without specialization in neonatology or pediatric intensive care was performed using MANOVA–Pilai trace, adjusted for a clinical scenario (hypoxemia, hypercapnia or normal blood gases), anxiety score, and self-reported experience with the iX5^®^ ventilator (Vyaire Medical, Chicago, Illinois, USA).

The sample size calculation was based on a study of physicians’ gaze tracking during newborn pain assessment, showing that gaze fixation on key facial regions occurred 80% of the time.^20^ Considering an alpha risk of 5% and an error of 10%, 62 participants would be required. Accounting for a 40% loss due to ocular signal non-acquisition, 87 participants should be included.

The statistical analysis was performed using Statistical Package for the Social Sciences (SPSS) 20.0 for Windows (IBM SPSS Statistics, Somers, NY).

## RESULTS

Among the 88 eligible physicians, 13 (15%) were excluded: two due to technical problems during the experiment, and 11 due to capture of ocular signal for less than 70% of the video analysis time. The 75 studied physicians were similar to those excluded in terms of age in years (33 [interquartile range – IQR] 30–39 vs. 29 [IQR 29–33]; p=0.305), gender (female: 84 vs. 100%; p=0.200), years since medical graduation (6 [IQR 4–14] vs. 4 [IQR 3–7]; p=0.672), residency in Pediatrics (88 vs. 85%; p=0.663), years since Pediatrics residency graduation (4.0 [IQR 1.0–13.0] vs. 3.0 [IQR 0.5–6.5]; p=0.889), fellowship in neonatology or pediatric intensive care (60 vs. 46%; p=0.377), years since fellowship graduation (7 [IQR 2–18] vs. 4 [IQR 3–16]; p=0.701), weekly hours of work in intensive care (48 [IQR 36–60] vs. 60 [IQR 48–60]; p=0.507), median anxiety score (4.0 [IQR 2–7] vs. 4 [IQR 1–10]; p=0.983) and self-reported experience with iX5 ventilator (83 vs. 69%; p=0.267).

**Figure 2:**
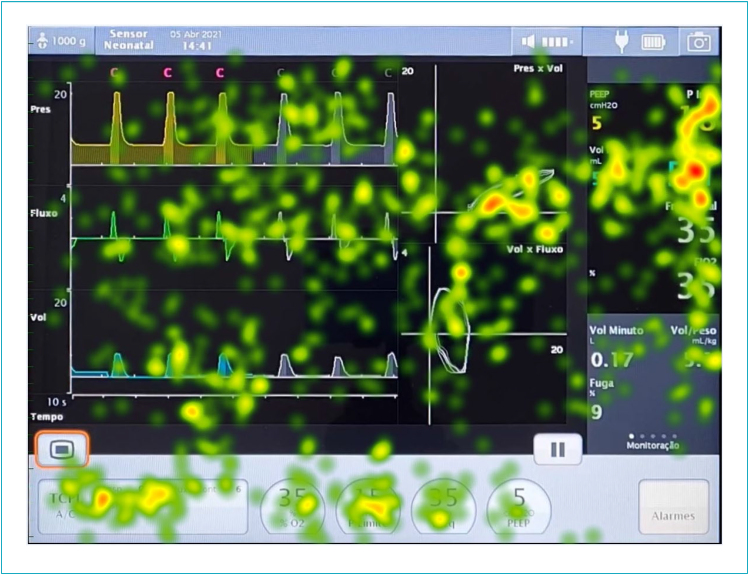
Heat map of visual fixation time in the areas of interest of the participants who evaluated the ventilator panel corresponding to the clinical case with normal blood gases: areas in green — shortest visual fixation time; areas in yellow — intermediate visual fixation time; areas in red — longest visual fixation time.

Source: Image generated by the eye-tracker system during the experimente.

In the 10 seconds in which the video was evaluated, acquisition of the ocular signal occurred during 95.2±4.7% of the time. During this period, the physicians performed 32.8±5.7 visual fixations and fixed their gaze for 8.0±1.2 seconds on the AOI. Table 2 shows physicians’ visual tracking outcomes data for each AOI. Participants exhibited a higher number of fixations and longer gaze duration on the curves and loops, followed by the monitored parameters and then the adjusted parameters. Figure 2 represents the heat map of a participant, indicating the time of visual fixations in the AOI.

**Table 2:** Gaze fixation, number of fixations, and duration of visual fixations (seconds) of the 75 participants on the areas of interest of the mechanical ventilator panels.

	Monitored parameters	Adjusted parameters	Curves and Loops
Gaze fixation [n (%)]	71 (95)	60 (80)	75 (100)
Number of fixations	8.03±5.98	5.40±5.00	14.72±7.64
Fixation time (seconds)	2.07±1.61	1.21±1.29	3.71±2.12

Source: Data obtained in the study.

Table 3 presents a comparison of physicians’ visual tracking outcomes across clinical scenarios. The number of physicians who fixated on the AOIs did not differ among the three scenarios. Adjusted for the anxiety score (<4 vs. >4) and the experience with the iX5^®^mechanical ventilator (Vyaire Medical, Chicago, Illinois, USA; yes vs. no), MANOVA showed no difference in the number of fixations on monitored parameters, adjusted parameters, and curves and loops, and in the time of visual fixations.

**Table 3:** Gaze fixation, number of fixations and time of visual fixations of the 75 participants on the monitored and adjusted parameters, and on the curves and loops, according to clinical scenarios.

		Case 1 Hypoxemia n=26	Case 2 Hypercapnia n=25	Case 3 Normal n=24	p-value
Gaze fixation [n (%)]					
	Monitored parameters	24 (92)	25 (100)	22 (92)	0.346
	Adjusted parameters	22 (85)	20 (80)	18 (75)	0.697
	Curves and loops	26 (100)	25 (100)	24 (100)	
Number of fixations					
	Monitored parameters	7.50±5.86	9.04±6.50	7.54±5.66	0.465*
	Adjusted parameters	5.62±4.90	6.12±4.99	4.42±5.17	
	Curves and loops	16.69±8.42	12.32±6.59	15.08±7.39	
Fixation time (seconds)					
	Monitored parameters	1.73±1.46	2.48±1.53	2.00±1.82	0.458*
	Adjusted parameters	1.15±1.05	1.56±1.42	0.92±1.35	
	Curves and loops	3.92±2.21	3.32±1.99	3.87±2.17	

* p-values refer to the combined analysis of monitored parameters, adjusted parameters, and curves/loops — MANOVA (Pilai Trace=0.079; F [6, 138]=0.946, p=0.465) and in the time of visual fixations (Pilai Trace=0.080; F [6, 138]=0.956, p=0.458).

Source: Data obtained in the study.

Table 4 shows the comparison of visual screening outcomes between physicians with and without specialization in neonatology and/or pediatric intensive care. All participants, regardless of whether they had completed their specialization, focused their attention on curves and loops. There was no difference in the number of physicians who fixed their gaze on the monitored and adjusted parameters, and curves and loops. Adjusted for clinical scenario (hypoxemia, hypercapnia, or normal blood gases), anxiety score (<4 vs. >4), and experience with the iX5^®^ mechanical ventilator (Vyaire Medical, Chicago, Illinois, USA; yes vs. no), MANOVA showed that there was a difference in the number of fixations in the monitored parameters, adjusted parameters and curves and loops. To identify the difference detected among the AOIs, pairwise comparisons were applied and revealed that physicians without specialization, compared to those with specialization, had a greater number of visual fixations in the adjusted parameters (p=0.004). Similarly, MANOVA showed a difference in the time of visual fixations in the monitored parameters, adjusted parameters and curves and loops between participants that had or had not completed a specialization in neonatology or pediatric intensive care. Pairwise comparisons showed that non-specialized physicians, compared to those with specialization, fixed their gaze longer on the adjusted parameters (p=0.002).

**Table 4:** Gaze fixation, number of fixations and duration of visual fixations of the 75 participants on the monitored and adjusted parameters, and on the curves and loops, according to specialization in neonatology or pediatric intensive care medicine.

Visual tracking variable		Specialization in neonatology or pediatric intensive care medicine		p-value
		Yes n=45	No n=30	
Gaze fixation [n (%)]				
	Monitored parameters	42 (93)	29 (97)	0.646
	Adjusted parameters	35 (78)	25 (83)	0.769
	Curves and loops	45 (100)	30 (100)	
Number of fixations				
	Monitored parameters-	7.49±6.01	8.83±5.95	0.012*
	Adjusted parameters	3.98±3.99	7.53±5.64	
	Curves and loops	15.78±7.84	13.13±7.17	
Fixation time (seconds)				
	Monitored parameters	2.02±1.66	2.13±1.57	0.018*
	Adjusted parameters	0.80±0.97	1.83±1.46	
	Curves and loops	4.02±2.26	3.23±1.81	

* p-values refer to the combined analysis of monitored parameters, adjusted parameters, and curves/loops" — MANOVA (Pilai Trace=0.147; F [3, 68]=3.914; p=0.012). (Pilai Trace=0.136; F [3, 68]=3.583; p=0.018).

Source: Data obtained in the study.

## DISCUSSION

This study evaluated physicians’ visual attention using eye-tracking while assessing a mechanical ventilator panel to determine whether to adjust ventilatory parameters. The descriptive results showed that physicians, in general, focused their gaze primarily on curves and loops, followed by monitored and adjusted parameters. The data showed no difference in participants’ visual attention according to the clinical scenario (hypoxemia, hypercapnia, or normal blood gases), likely due to the small sample size for this secondary analysis. When comparing physicians with and without specialization in neonatology or pediatric intensive care, there was no difference in the number of participants who fixated on the three areas of interest. However, non-specialists exhibited a higher number of fixations and longer gaze duration on the adjusted parameters than specialists.

The increased visual attention to curves and loops can be attributed to their higher cognitive processing demands compared to numerical parameters, which are interpreted more objectively and quickly. Additionally, since physicians assessed a video of the ventilator panel, the curves and loops presented dynamic stimuli, potentially drawing greater interest than the static monitored and adjusted numerical parameters. Dynamic stimuli, compared to static stimuli, when expressing equivalent information, attract more visual attention from the observer and may indicate a greater cognitive challenge.^21^ A similar finding was reported in a study where physicians and nurses performed positive pressure ventilation in a neonatal resuscitation simulation scenario with monitored mannequins. During the ventilation, participants fixed their gaze on exhibited curves for 33% of the total time, while numerical parameters were fixated on for 20% of the time.^22^ In another study on patient extubation, only 54% of the physicians focused on the respiratory rate during the procedure, 59% focused on tidal volume, 64% on peak pressure, 64% on carbon dioxide levels, 18% on temperature, 59% on blood pressure, and 68% on heart rate. However, nearly all physicians focused on pressure and volume curves.^23^

The analysis of physicians’ visual tracking when evaluating a ventilator panel in cases of hypoxemia, hypercapnia, or normal blood gases revealed no differences in the number of physicians who fixated on curves and loops, monitored parameters, or adjusted parameters. In all three scenarios, 100% of physicians fixated on curves and loops, over 90% on monitored parameters, and more than 75% on adjusted parameters. Similarly, no differences were observed in the number or duration of visual fixations across these areas. These findings suggest a pattern in how physicians assess ventilator panels, regardless of the clinical scenario. The small sample size may also have contributed to the failure to observe differences in the focus of visual attention between participants who evaluated the different ventilator panels.

In the analysis of physicians’ visual tracking when evaluating the ventilator panel based on their specialization in neonatology or pediatric intensive care medicine, after adjusting for the clinical scenario, anxiety score, and experience with the iX5^®^ ventilator, differences were observed in the number and duration of visual fixations. Physicians without specialization had a higher number of visual fixations and spent more time focusing on the adjusted parameters compared to specialists. This may reflect their lower level of training in extracting and interpreting the information provided by the monitored parameters and curves and loops. Another possible explanation is that individuals with greater experience recognize patterns more quickly and therefore stop looking sooner.

Our study had some limitations, primarily related to sample size, as 15% of the 88 eligible physicians were excluded. However, the excluded participants had demographic characteristics similar to those included in the analysis. Additionally, the limited sample size precluded a more detailed assessment of differences in visual attention to specific ventilator parameters across different clinical scenarios or between physicians with and without specialization in neonatology or pediatric intensive care medicine. Another limitation relates to the study setting. Ventilator panels were evaluated using video recordings displayed on a computer screen in a controlled environment, which did not replicate the auditory and visual distractions typical of a NICU. The NICU environment is also more stressful, as clinicians are responsible for correctly managing the ventilator, which can directly affect patient survival. Replicating this study in a real NICU, with healthcare professionals responsible for patient care, would therefore be valuable for comparison. It should also be noted that the physician's position in relation to the ventilator differed in the experimental paradigm compared to real-life NICU conditions. Furthermore, we used only the iX5 fan in the experiment, so the results may differ with other interfaces. Therefore, the generalization of the results should consider the fact that 60% of the physicians had specialization in neonatology or pediatric intensive care medicine, and that the experiment was conducted using the iX5 ventilator.

Our study has several strengths. To our knowledge, it is the first to evaluate physicians’ visual attention when assessing a mechanical ventilator panel and making decisions about ventilatory adjustments in different clinical scenarios. The use of the Tobii TX300 eye tracker, a highly precise and reliable device, ensured robust data collection, capturing ocular signals in 95% of the experiment time. Additionally, our analyses comparing visual tracking outcomes across clinical scenarios and between physicians with and without specialization in neonatology or pediatric intensive care were adjusted for participants’ anxiety levels and prior experience with the ventilator, enhancing the reliability of our findings.

Our study may contribute to enhancing physician training in ventilatory management for newborns on mechanical ventilation, particularly by emphasizing the interpretation of curves and loops when adjusting parameters. Additionally, our findings could inform improvements in the layout of ventilator panel information, optimizing usability and enhancing the efficiency and safety of mechanical ventilation. Future research should explore physicians’ visual attention when assessing ventilator panels and deciding on parameter adjustments in bedside settings and with different ventilator models. Studies with larger sample sizes could provide more detailed insights into visual tracking by analyzing individual ventilatory parameters rather than grouped data. Furthermore, investigating physicians’ gaze trajectories until the moment of maximum pupillary dilation — an indicator of peak cognitive load — could offer valuable insights into the decision-making process.^17-19,24-26^

In conclusion, our findings indicate that during the decision-making process, physicians evaluating mechanical ventilator panels focus their attention mostly on curves and loops, followed by monitored and adjusted parameters. Visual attention did not differ when assessing ventilator panels in clinical scenarios of hypoxemia, hypercapnia, or normal blood gases. However, physicians without specialization in neonatology or pediatric intensive care medicine, compared to specialists, showed greater attention to the adjusted parameters.

## Data Availability

The database that originated the article is available with a corresponding autor.
